# Vaginal Swelling After Intercourse: A Case Report

**DOI:** 10.5811/cpcem.2022.2.55284

**Published:** 2022-04-25

**Authors:** Michael J. Bono, Dylan M. Woolum, A. Shirley Jones, Francis L. Counselman

**Affiliations:** * Eastern Virginia Medical School, Department of Emergency Medicine, Norfolk, Virginia; † Emergency Physicians of Tidewater, Norfolk, Virginia

**Keywords:** vaginal swelling, vulvar hematoma, labia majora, labia minora, case report

## Abstract

**Introduction:**

A vulvar hematoma is a hemorrhagic fluid collection in the female external genitalia. The majority occur as an obstetrical complication, especially during labor. Non-obstetrical vulvar hematomas are usually the result of trauma, with coitus being the most common etiology.

**Case Report:**

We present the case of a 25-year-old woman with significant vaginal pain and swelling after vigorous sexual intercourse. She exhibited tenderness and swelling of the left labia majora and minora. The differential diagnosis included bleeding, abscess, and deep venous thrombosis. Laboratory studies were normal and computed tomography of the pelvis indicated the swelling was most likely due to blood. The patient was taken to the operating room, and approximately 150 cubic centimeters of clot was evacuated. The patient had an uneventful recovery and was discharged home the next day.

**Conclusion:**

This case illustrates the unique presentation and challenges in making the diagnosis of vulvar hematoma.

## INTRODUCTION

We present the case of a 25-year-old woman who presented to the emergency department (ED) with the complaint of increasing pain and swelling of the left side of her vagina. She admitted to frequent and vigorous sexual intercourse the day prior. Physical exam was remarkable for a swollen, dark, and tender left labia majora and minora. Laboratory studies were normal and computed tomography imaging of the pelvis revealed the swelling was most likely due to blood. The patient was taken to the operating room (OR) where 150 cubic centimeters (cc) of clot was evacuated and a bleeding arteriole was ligated. She was discharged the day after surgery and had an uneventful recovery. We discuss vulvar hematoma, an uncommon diagnosis, its presentation, and management.

## CASE REPORT

A 25-year-old woman presented to the ED complaining of left-sided vaginal pain and swelling. She stated she had woken up approximately five hours earlier with these symptoms, which had been progressing. She described the pain as severe and throbbing. She stated she was unable to sit comfortably and that it was painful to walk. The patient reported having five episodes of sexual intercourse over a two-hour period the day prior; afterwards, she applied ice to the area. She denied abdominal pain, vaginal bleeding, or discharge. The patient was in good health otherwise and denied any medical problems, including sickle cell disease or bleeding disorders. She was not on any medications and denied drug allergies.

Physical exam revealed a young woman in mild distress secondary to severe pain. Vital signs revealed a pulse of 96 beats per minute, respiratory rate of 14 breaths per minute, blood pressure of 123/86 millimeters of mercury (mm Hg), temperature of 97° Fahrenheit (36.1° Celsius), and 100% oxygen saturation on room air. Examination of the heart, lungs, and abdomen was normal. Genitourinary exam revealed marked swelling of the left labia majora and minora. The swollen area was dark, firm, and tender to palpation (Image). Speculum exam was deferred due to the significant amount of pain and swelling.

We were concerned for hematoma primarily, but also considered abscess or a deep venous thrombosis (DVT). An intravenous (IV) line was established and the patient was administered morphine four milligrams (mg) and ondansetron four mg IV for pain. She required repeated dosing in the ED for adequate pain control. Laboratory studies were sent for a complete blood count (CBC), basic metabolic profile (BMP), coagulation studies, D-dimer, urinalysis, and urine pregnancy test. Results included a normal CBC, BMP, coagulation studies, D-dimer, and urinalysis. The urine pregnancy test was negative.

The gynecology service was consulted; they also were unable to perform a speculum exam. After discussion, it was mutually agreed to image the patient with a computed tomography (CT) of the pelvis. The CT of the pelvis with IV contrast showed an enlarged left labial mass-like lesion, most likely due to focal hemorrhage or hematoma, but no definite involvement of the base of the bladder or urethra. There was a 6.6 x 4.8 cm hyperdense ovoid focus within the left labial region. Given this finding, and that the swelling appeared to be expanding, the patient was taken to the OR. A two-centimeter incision was made on the mucosal surface of the labia minora and approximately 150 cc of clot was evacuated. Also identified was an area of arterial bleeding, which was ligated with 3.0 Vicryl suture (Ethicon, Inc., Somerville, NJ).

On the first postoperative day, the patient felt much improved, with significant reduction in the swelling and pain. Her hemoglobin and hematocrit were unchanged from the day prior. She was discharged home with a diagnosis of vulvar hematoma, with instructions to follow up in one week and for pelvic rest for one month.

**CPC-EM Capsule**

What do we already know about this clinical entity?

*The majority of vulvar hematomas are a result of obstetrical complications. The majority of non-obstetrical vulvar hematomas are related to trauma, including sexual assault and coitus*.

What makes this presentation of disease reportable?

*This is an excellent example of a non-obstetrical vulvar hematoma due to trauma. The physical exam findings are classic for this condition, and emphasize the importance of appropriate imaging*.

What is the major learning point?

*The role of ultrasound and computed tomography, the pros and cons of each, in evaluating a vulvar hematoma*.

How might this improve emergency medicine practice?

*This case should broaden the differential diagnosis when evaluating a woman complaining of vulvar pain and swelling. The appropriate evaluation and management strategy are reviewed*.

## DISCUSSION

The vulva comprises the external sex organs made of smooth muscle and connective tissue that surrounds the vaginal introitus and urethra. A vulvar hematoma is a hemorrhagic fluid collection within these connective tissues.^1^, ^2^ The vulva is well vascularized by the internal pudendal artery. The pudendal artery is a branch off the internal iliac artery, and it supplies the majority of the perineum. Once through the pudendal canal located in the ischioanal fossa, the pudendal artery subdivides into smaller branches, including the perineal artery, which provides vascular flow to the external genitalia.^2^, ^3^ The pudendal artery, along with its branches, accounts for the majority of vulvar hematomas.^2^ Less common culprit lesions may include injury to the internal iliac artery, given its more proximal origin, or venous bleeding.^4^

Obstetric complications account for most vulvar hematomas, with an incidence of 1–2 per 1000 deliveries.^5^ Most of these occur during labor. Outside the pregnant population, vulvar hematomas have an incidence of 3.7% and represent only 0.8% of all gynecologic admissions.^6^ The majority of non-obstetric vulvar hematomas are related to various traumatic insults, including saddle injuries, falling from a height, sexual assault, and coitus.^6^, ^7^,^8^ If there is no history of trauma then a spontaneous vessel rupture, such as a pseudoaneurysm of the internal iliac artery or pudendal artery, should be considered.^2^ As in our case, coitus is the most common non-obstetric etiology of a vulvar hematoma. 1 Hematoma formation is suspected to be due to direct compression of the labial and vaginal soft tissues against adjacent pelvic bone resulting in laceration of underlying vasculature. 8

Common presentations for a vulvar hematoma include perineal pain and unilateral swelling. Non-obstetric vulvar hematomas follow a bimodal age distribution. It is more common during childhood or early adolescence (because the labia majora is less developed) and in postmenopausal women as a result of hypoestrogenism, making the vulva more prone to injury.^7^ For unknown anatomic reasons, the majority of vulvar hematomas are right sided (70%).^7^ When significant swelling is present, pain may be accompanied by urological manifesta-tions, including dribbling or urinary retention secondary to urethral obstruction.^4^ Importantly, if hematoma formation is secondary to aggressive coitus, clinicians should privately discuss with the patient any concerns for sexual assault.

Physical examination involves visual inspection of the vulva and vagina. Although pain and swelling may limit this exam, the vaginal canal should be directly visualized to exclude mucosal laceration. In the setting of trauma, associated injuries such as fractures must be considered.^8^ Due to inflammation and edema of surrounding soft tissue, erythema and tenderness may be mistaken for Bartholin’s gland abscess or folliculitis.^1^, ^8^, ^9^ Additional considerations in the differential diagnosis include coagulopathies, varicosities, contact (condom) dermatitis, angioedema, DVT, or carcinoma.

Most patients with clinically significant hematomas should have a CBC performed to evaluate for anemia. Further coagulation studies or blood screening and cross-matching may be necessary, should bleeding be significant. A variety of imaging modalities can be used in diagnosing and monitoring vulvar hematomas, including CT, ultrasound, and magnetic resonance imaging.^10^ Transperineal sonography, using 7.5- and 10-megahertz transducers, has been shown to be effective in depicting the size of vulvar hematomas. 11 Ultrasound has the advantages of being able to be performed at the bedside, a lack of ionizing radiation to the genital region, and lower cost. A CT with use of IV contrast can accurately identify the size of the fluid collection and the presence of extravasation. This is at the expense of higher cost and exposure of the genital region to ionizing radiation. Magnetic resonance imaging has long been used for the imaging of vulvar malignancies, due to its high soft tissue contrast and excellent anatomic detail, without ionizing radiation. 12 However, the increased cost and time delay must be considered.

There is no consensus for the management of vulvar hematomas. In practice, smaller hematomas with gradual onset and minimal pain are often managed conservatively with ice packs, analgesia, and bed rest.^1^ Appropriate monitoring and follow-up are warranted to avoid complications such as pressure necrosis or infection.^10^ Larger hematomas that are either expanding or have associated pressure necrosis or pelvic injury, and are greater than 10 cm in size, or have associated hemodynamic instability, must be recognized and managed early.^6^ Conservative management of large hematomas has been found to be associated with a longer period of hospitalization, and a greater need for antibiotics and blood transfusion.^7^, ^13^ Management includes surgery or directed arterial embolization in consultation with interventional radiology. For our patient, given her degree of pain and the progressive swelling in the ED, the patient was taken to the OR for hematoma evacuation. The culprit bleeding artery was directly visualized and ligated.

## CONCLUSION

Although mortality is rarely associated with a vulvar hematoma, its potential morbidity makes this one diagnosis that emergency physicians must consider when evaluating a woman with a perineal complaint. The potential complications include necrosis, infection, neurologic compromise, urinary obstruction, and hemo-dynamic instability. While most vulvar hematomas are related to obstetric complications, they may also occur spontaneously or from local trauma, such as coitus.

## Figures and Tables

**Image:**
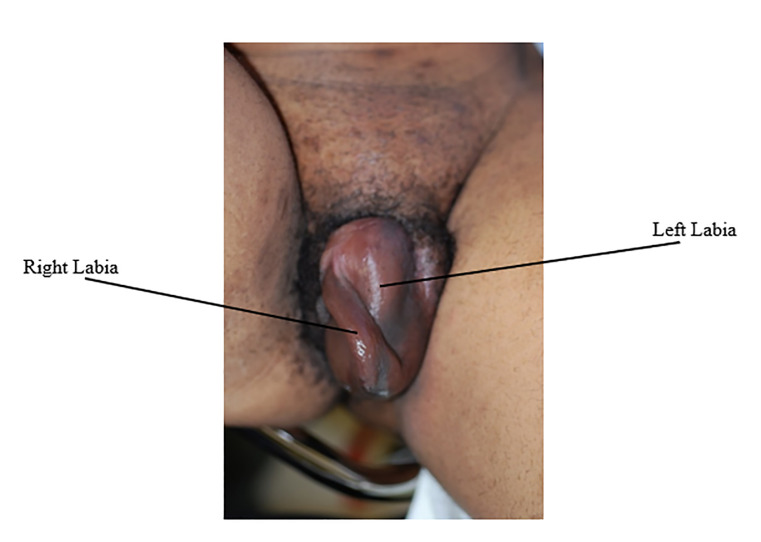
Swollen, darkened left labia majora in patient with vulvar hematoma.
